# Kidney sparing giant retroperitoneal liposarcoma: Case report and literature review

**DOI:** 10.1016/j.ijscr.2019.02.008

**Published:** 2019-02-21

**Authors:** Masjensen Argadjendra, Rudi Napitupulu, Redemptus Yudadi, Sakti Hoetama, Heru Seno Wibowo

**Affiliations:** a Hang Tuah University, Surabaya, Indonesia; b Department of Digestive Surgery, Dr Ramelan Navy Hospital Surabaya, Surabaya, Indonesia; c Department of Urological Surgery, Dr Ramelan Navy Hospital, Surabaya, Indonesia

**Keywords:** Retroperitoneal liposarcoma, Well-differentiated, Nephrectomy

## Abstract

**Introduction:**

Liposarcoma is the most common variant of soft tissue sarcoma. It often originates in retroperitoneum or perirenal fat. Early diagnosis is extremely difficult due to its location in the retroperitoneum that allows for room for growth. Symptoms would only occur when the tumor becomes very large and/or invades into adjacent organs.

**Presentation of case:**

We report a case of a 30 years old woman who underwent surgical resection for a well-differentiated liposarcoma. CT scan revealed that the mass invaded the left perirenal fascia and displaced the descending colon, pancreas, and duodenum. Complete resection of tumour masses were performed and we opted against nephrectomy due to the tumor's histologic type, the patient's young age, and increased morbidity.

**Discussion:**

Treatment for Retroperitoneal Liposarcoma involves R0 resection. In cases where the tumor invades into a nearby organ, it is necessary to weigh the benefit of free margin resection against the adversity of medical complication and quality of life loss.

**Conclussion:**

Due to its rate of recurrence, a life long follow up and routine assessment of intraabdominal mass should be integrated into clinical practice in liposarcoma patients.

## Highlights

• Soft tissue sarcoma accounts for less than 1% of all malignant tumors.
• Early diagnosis of Retroperitoneal Liposarcoma is extremely difficult due to its location and early onset lack of symptoms.
• Complete surgical excision remains the mainstay of treatment.
• Complete resection of invaded organs must be weighed for its recurrence or patient adversity.
• Retroperitoneal Liposarcoma of well-differentiated subtypes has a good prognosis and low metastatic potential.

## 1 Introduction

Soft tissue sarcoma (STS) accounts for <1% of all malignant tumors. Liposarcoma is the most common variant and accounts for 20% of all STS [1]. The World Health Organization Classification classified it into 5 subtypes histologically: i) well-differentiated; ii) myxoid, iii) round cell, iv) pleomorphic, and v) dedifferentiated [2]. Liposarcoma may occur wherever fat is present with approximately 12–40% originating from the retroperitoneum and 35% from the perirenal fat [3]. Notwithstanding its rarity, with an incidence of 2.5/1,000,000, early diagnosis of Retroperitoneal Liposarcoma (RPS) is extremely difficult owing to its location in the retroperitoneum since symptoms would only appear when the tumor becomes very large and/or invades into adjacent organs. Indeed, in the largest series of prospectively followed RPS, 94% of the tumor exceeds 5 cm in diameter and 60% exceeds 10 cm [3]. The mainstay of treatment for this condition is complete surgical excision. Resection of invaded organs is still a controversial topic since the danger of multi-organ resections may outweigh the possibility of recurrence [4,5]. This work has been reported in line with the SCARE criteria [6].

## 2 Presentation of case

**Fig. 1:**
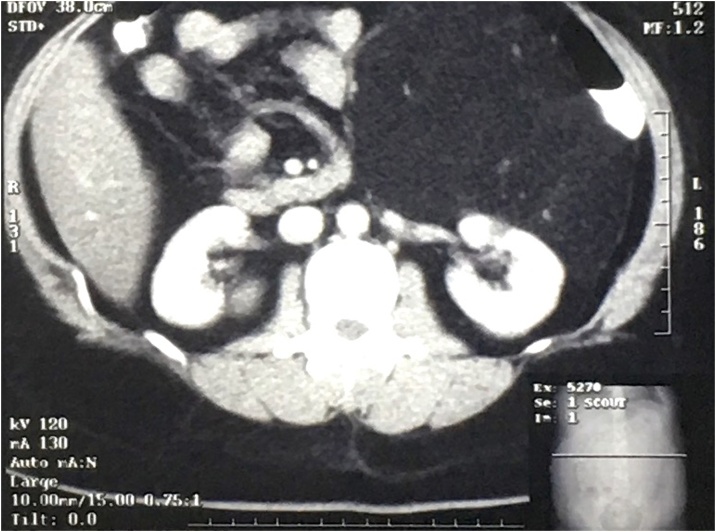
XXX.

**Fig. 2:**
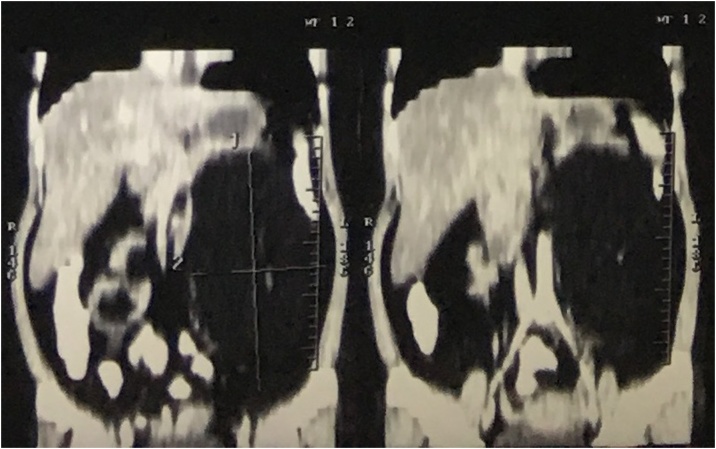
XXX.

A 30-year-old woman presented with a history of abdominal pain and distension. System reviews were otherwise normal. There were no significant past medical history or history of familial diseases. Physical examination revealed palpable abdominal mass at the Left Upper & Lower Quadrant measuring around 20 cm with a rubbery consistency. Laboratory examinations of complete blood counts, urine tests, and tumor markers were otherwise normal. CT scan of the abdomen and pelvis revealed a hypodense mass (−89 to −94) with a clear border. The mass invaded the left perirenal fascia and displaces the descending colon, pancreas, and duodenum (Fig. 1, Fig. 2). Percutaneous biopsy of the lesion was not performed due to the risk of tumor seeding.

The patient underwent an exploratory laparotomy which revealed a bulky lesion originating from the left retroperitoneal region. The lesion adheres to the anterior and inferior portion of the left kidney and left psoas major muscle with infiltration of the left renal parenchyma. We opted to preserve the kidney owing to the patient's young age. No lymphadenopathies, hepatic nodules, and urethral infiltrations were observed.

**Fig. 3:**
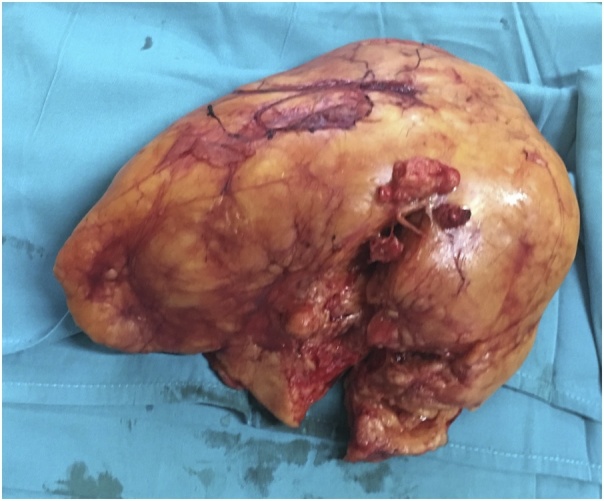
XXX.

**Fig. 4:**
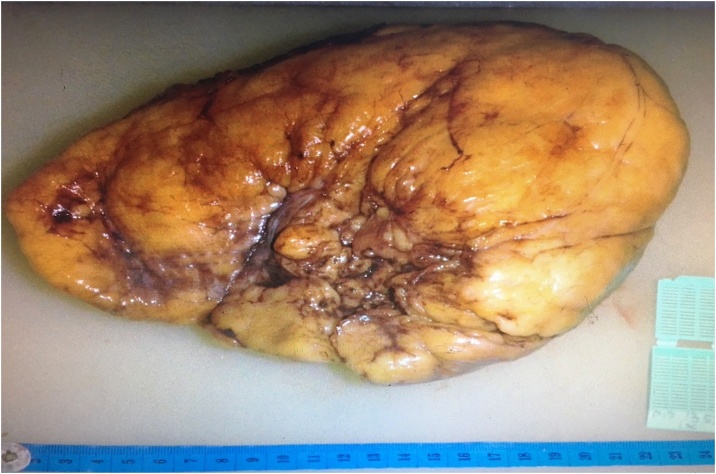
XXX.

The mass measured 21 × 16,5 × 10 cm and with heterogenous yellowish mass appearance (Fig. 3, Fig. 4). Hematoxylin and eosin staining of the tissue showed adipocyte cellular proliferation with atypical oval-round nuclei, gross chromatin, and clear cytoplasm – all of which suggests well-differentiated liposarcoma.

The patient's postoperative course was uneventful and she was discharged on the 4th postoperative day. The patient is disease free at 12 months of follow up. Tumor recurrence from repeat abdominal and pelvic CT scans at 6 and 12 months was not observed.

## 3 Discussion

Retroperitoneal liposarcoma (RPS) is a rare disease with poor 5-year survival rate even with complete removal of the tumor. Following R0 resection, a well-differentiated sub-types has a 5-year survival rate of 90% while for pleomorphic subtypes the rate is only 30–50% [7,8]. Furthermore, owing to retroperitoneal space's expandable characteristic, retroperitoneal liposarcoma would first grow without compressing vital organs in its early clinical course and would only show symptoms when they grow too large and compresses or invades adjacent organs. When symptoms do occur, they tend to be non-specific and include abdominal pain/fullness, flank pain, early satiety, lower extremity swelling, or pain. Local invasion of compression of retroperitoneal structures may present as neurological, musculoskeletal, and obstructive urinary/bowel symptoms. No significant laboratory abnormalities are observed in the disease’s early stage and diagnosis is often made only when the tumor has grown to a large size.

Mortality from RPS often results from local recurrence due to incomplete resection of tumor mass – often from difficulty in differentiating the liposarcoma from adjacent normal fat and from the absence of an anatomically evident vascular-lymphatic peduncle that makes it hard to obtain safe margin [9]. RPS frequently recurs within 6 months to 2 years after the initial surgical resection and it has a rapid growth pattern with mean tumor volume doubling time of around 100 days [10]. Prognosis from RPS following complete resection depends on its histologic type. The 5-year survival rate for well-differentiated subtypes is 90%, dedifferentiated at 75%, and myxoid/round cell at 60–90%, and pleomorphic subtypes at 30–50% [3].

While treatment for RPS involves R0 resection, tumor burden and nephrectomy are not associated with disease specific survival [10]. In cases where the tumor invades into a nearby organ, such as the left kidney with our patient, it is necessary to weigh the benefit of free margin resection against the adversity of medical complication and quality of life loss. Despite invasion to left renal parenchyma and the possibility of future recurrence from that site, we elected not to perform nephrectomy due to the tumor’s histologic type, patient's young age, and increased morbidity from resection. Furthermore, the pathological report revealed that the patient's tumor is of the well-differentiated liposarcoma subtype which is characterized by its local aggressiveness and low metastatic potential. Prognosis of well-differentiated RPS is generally excellent where the 5 year survival rate is 90% with 5 year local recurrence rate at approximately 50%. In contrast, RPS’s other histologic types have lower 5 years survival rate and necessitates a more aggressive approach [3,11]. Our patient is disease free at 12 months following resection. In a study involving 228 patients with retroperitoneal liposarcoma undergoing surgery, patients with combined organ surgeries has a lower 10-year survival rate of 26% as opposed to 35% rate for patients not requiring multi-organ resection [12]. Therefore, this study suggest that nephrectomy must only be performed if it is required to accomplish a complete gross resection and if the histologic subtypes is not of the well-differentiated type.

## 4 Conclusion

Once diagnosed with liposarcoma, a life-long follow-up and routine assessment of intraabdominal mass should be integrated into clinical practice due to the high rate of recurrence. Future sign of recurrence should be managed with surgical excision and the necessity of organ resection in case of organ invasion should be carefully balanced with the patient's quality of life following surgery.

## Conflicts of interest

All authors declare that they have not any conflict of interest.

## Funding

The authors declare there are not any sponsors involvement.

## Ethical approval

The authors declare that all procedures followed were in accordance with the ethical standards of the responsible committee on human experimentation (institutional and national). Informed consent was obtained from the patient for being included in the study.

## Consent

Authors declare that they have obtained written informed consent from the patient for publication of this case report and accompanying images. A copy of the written consent is available for review by the Editor-in-Chief of this journal on request.

## Author contribution

Masjensen Argadjendra concept or design, data collection, data analysis or interpretation, writing the paper.

Rudi Napitupulu contributor, study concept or design.

Redemptus Yudadi contributor, study concept or design.

Sakti Hoetama contributor, study concept or design, and reviewer.

Heru Seno Wibowo study concept or design, data collection, data analysis, or interpretation and reviewer.

## Registration of Research Studies

None.

## Guarantor

Masjensen Argadjendra.

Rudi Napitupulu.

Redemptus Yudadi.

Sakti Hoetama.

Heru Seno Wibowo.

## Provenance and peer review

Not commissioned, externally peer-reviewed.
